# Effect of Testing Conditions on Low-Cycle Fatigue Durability of Pre-Strained S420M Steel Specimens

**DOI:** 10.3390/ma17081833

**Published:** 2024-04-16

**Authors:** Stanisław Mroziński, Michał Piotrowski, Halina Egner

**Affiliations:** 1 Faculty of Mechanical Engineering, Bydgoszcz University of Science and Technology, Al. Kaliskiego 7, 85-796 Bydgoszcz, Poland; stmpkm@utp.edu.pl (S.M.); michal.piotrowski@pbs.edu.pl (M.P.); 2 Faculty of Mechanical Engineering, Cracow University of Technology, Al. Jana Pawla II 37, 31-864 Kraków, Poland

**Keywords:** low-cycle fatigue, durability prediction, experimental testing, pre-strain, cyclic properties

## Abstract

S420M steel subjected to strain-controlled low-cycle fatigue does not exhibit a period of cyclic properties stabilization. The maximum stress on a cycle continuously drops until fracture. For this reason, it is difficult to plan experimental research for different types of control in such a way that their results can be considered comparable. The aim of this paper is to present and discuss the results of tests conducted in various conditions of low-cycle fatigue of S420M steel specimens, both undeformed and pre-strained. In both loading conditions, after initial deformation, a significant change in the cyclic properties of steel described by the parameters of the hysteresis loop was observed. Also, the fatigue life of the pre-strained specimens appeared to be different from unstrained specimens and was affected by the test loading conditions. The reduction in life under controlled stress conditions was attributed to the increase in the extent of plastic deformation and stress and the occurrence of creep.

## 1. Introduction

Structural elements of many technical devices, such as elements of power plants, bridges, and wind farms, may be subjected to occasional significant static loads during operation, resulting, for example, from improper start-up, assembly, or shutdown due to seismic events. The effects of such unforeseen events (emergencies) in the form of initial deformations are usually disregarded in fatigue calculations, which are most often based on the data determined experimentally using as-received materials [1,2,3,4,5,6]. However, to properly design important technical objects that may be subject to occasional unforeseen events, it is necessary to have detailed knowledge about the construction material response to possible additional deformation, which may affect the course of material stabilization and material data used in fatigue calculations.

The impact of the initial deformation on the basic strength properties of engineering materials was discussed in many research papers in relation to both static and fatigue loading conditions. In papers [7,8,9], a beneficial effect of the initial deformation on the strength characteristics obtained as a result of carrying out static tensile tests was demonstrated for precipitation-strengthened stainless steel SUH660 [7], complex phase steel CP800 [8], and austenitic stainless steel AISI 304L [9]. However, such an improvement in basic strength parameters due to initial deformation was not observed for S420M steel [10].

As described in the literature, tests of the impact of initial deformation on the fatigue life were carried out under various cyclic loading conditions. Mnif et al. in [11] performed strain-controlled torsion fatigue tests ($\tau_{at}$ = const.) of brass alloy specimens and found that initial deformation results in a slight increase in fatigue life. Such an increase in the fatigue life and fatigue limit of specimens made of sheet metal used for car bodies, as a result of 10% of initial strain, was also noted in [12]. They carried out fatigue tests under conditions of flat bending and controlled strain ($\varepsilon_{at}$ = const.). On the other hand, a slight reduction in the fatigue life of SUH660 austenitic steel specimens after 10% pre-strain was found in [7] under the conditions of rotating–bending fatigue under controlled stress ($\sigma_{a}$ = const.). The authors of [13] investigated the pre-strain-related changes in the fatigue limit of 0.1% (annealed) and 0.5% (quenched and tempered) carbon steel by using a rotating bending fatigue test. The changes were correlated with the surface parameters. Increasing surface hardness improved the fatigue limit of 0.1% carbon steel, while pre-cracks generated on the surface decreased the fatigue limit in the case of 0.5% carbon steel. The authors of [14] conducted fatigue tests of 27MnCr5 steel samples in three loading conditions: tension, bending, and torsion. The results were analyzed to investigate the influence of pre-strain on fatigue strength. All investigations showed an increase in fatigue strength caused by strain hardening.

Most of the research concerning the effect of initial deformation on the fatigue life takes into account tension–compression tests, either under conditions of controlled strain ($\varepsilon_{at}$ = const.) [15,16,17,18] or stress ($\sigma_{a}$ = const.) [19,20,21,22]. The authors of [15] observed that the initial deformation reduces fatigue life and leads to a new state of stabilization of the C45 steel. The authors of [16] investigated the fatigue behavior of pre-deformed TRIP 780 steel specimens. The authors found that initial deformation followed by tempering increases the durability of steel for cyclic strain amplitudes larger than 0.004. The authors of [17] analyzed the effect of initial strains of TRIP780 multiphase steel specimens applied at different temperatures (−20 °C, 0, and 80 °C). A slight dependence of fatigue life on the initial deformation temperature was found. Mroziński et al. in [10] considered the strain-controlled fatigue tests of S420M steel specimens after initial deformation and observed a slight increase in the fatigue life of pre-strained specimens. The largest increase in fatigue life was detected at the lowest strain levels. On the other hand, the authors of [18] analyzed the influence of initial strains on the durability of the 7050-T6 aluminum alloy. Based on the examination of the strain–life and energy–life graphs, it was found that increasing initial deformation decreases the fatigue life. The authors of [23] analyzed the influence of both cyclic and monotonic pre-strains of similar magnitude on the cyclic behavior of pure copper and 316L stainless steel. It appeared that the impact of cyclic and monotonic pre-strains was similar for the investigated range of plastic strain amplitudes. In both cases, strain-controlled fatigue tests on pre-strained specimens revealed reduced fatigue life in comparison with as-received specimens. Whittaker and Evans [24] investigated the effect of pre-strain on the fatigue properties of Ti834 in the strain-controlled tests. They discovered that at low pre-strain levels, the mechanical properties change minimally, and durability is not significantly affected. On the other hand, 8% or more pre-strain significantly influences the fatigue properties of Ti834 and reduces durability. The authors of [25] investigated the pre-strain inherited memory effect by experimentally testing three FCC metallic materials: OFHC pure copper, nickel–chromium alloy, and AISI 316L stainless steel. Tensile pre-strain levels corresponded to different strain-hardening stages, and a wide range of strain amplitudes was considered. Three memory regions have been distinguished depending on the cyclic plastic strain amplitude.

For stress-controlled fatigue tests, the initial straining most often causes a reduction in the fatigue life. Yang and Wang [19] reported the influence of pre-strain on the reduction in durability and change in cyclic properties of high-strength spring steel specimens. The impact of the initial strain on the durability depended on both the magnitude of the initial strain and the amplitude of the variable load applied after it. The influence of the magnitude of pre-strain on the reduction in durability was confirmed in [8]. The reduction in fatigue life due to initial strains was also found in [7,20,21]. In these studies, it was found that the most significant reduction in durability takes place at the level of the fatigue limit. The authors of [22], based on tests of specimens made of austenitic steel Z2CN18.10, found that tensile pre-strain improves durability, while for compressive pre-strain, the fatigue life is reduced.

Based on the analysis of numerous literature reports (cf also [26,27,28,29,30,31,32] and many others), it can be concluded that the impact of initial deformations on the fatigue life largely depends on the load conditions occurring after the initial deformation. In the case of stress-controlled cycles ($\sigma_{a}$ = const.), a decrease in fatigue life is most often observed. In the case of strain-controlled conditions ($\varepsilon_{at}$ = const.), the durability of the specimens may depend also on other factors, such as the type of material, amount of deformation, etc. 

The research problem undertaken in the present paper is the quantitative assessment of the impact of loading conditions applied after initial deformation on the fatigue life of S420M steel specimens. S420M steel is very often used for welded elements that are subjected to very high static and fatigue loads. Typical applications for S420M steel are bridges, viaducts, long-span structures, high-rise buildings, and other responsible engineering structures. Due to frequent cases of overloads of these types of objects, tests are necessary to investigate how such events influence the durability of S420M steel elements. The experiments determining the basic strength parameters of both as-received and pre-strained S420M steel specimens in a static tensile test were already performed and described in [10]. It was shown that a pre-strain of S420M steel specimens of as much as 33% of the total elongation ($A_{5}$) does not reduce the basic strength parameters determined in the static tensile test. In the present research, low-cycle fatigue tests were carried out under conditions of controlled strain ($\varepsilon_{at}$ = const.) and controlled stress ($\sigma_{a}$ = const.). The analysis of hysteresis loops parameters and metallographic microstructure observations were performed to formulate the conclusions. 

## 2. Materials and Methods

### 2.1. Experimental Methods

#### 2.1.1. Test Specimens

The test samples were cut out of a S420M steel sheet with a thickness of 30 mm. Table 1 summarizes the chemical composition of S420M steel.

**Table 1:** Chemical composition of S420M steel (wt %).

Fe	C	Si	Mn	P	Cr	Al	Nb	Ti	V	W
98.0	0.125	0.215	1.45	0.0135	0.0208	0.0268	0.0288	0.013	0.0519	0.0150

**Figure 1:**
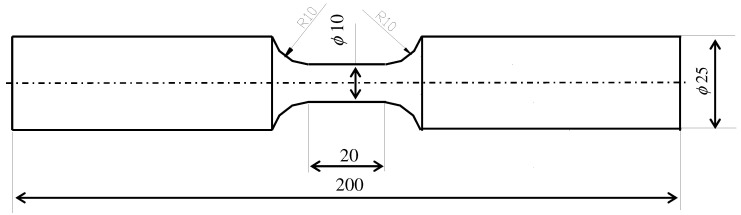
Test specimen (dimensions given in mm).

Specimens were prepared according to the requirements of standards [33,34,35] (see Figure 1).

After measuring the specimen diameters, the $R_{a}$ roughness measurements of the gauge part were carried out. Measurements were performed on a MarSurf XR 20 profilographometer, on three randomly selected specimens. The mean value $R_{a}$ = 0.59 was obtained.

#### 2.1.2. Low-Cycle Fatigue Tests

**Figure 2:**
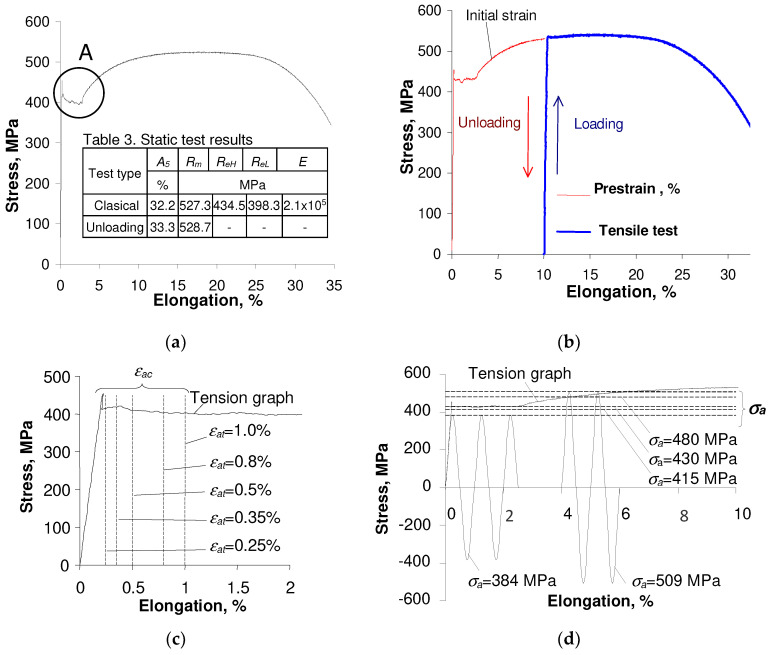
Tensile diagrams used for fatigue tests strain amplitude determination: (**a**) monotonic tests; (**b**) tension with unloading; (**c**) strain amplitude levels $\varepsilon_{at}$ adopted in tests (enlarged section A of stress-strain curve shown in (**a**); (**d**) stress amplitude levels $\sigma_{a}$.

During the low-cycle tests, both undeformed specimens (as-received specimens) and pre-strained specimens (ε=10%) were used. Fatigue tests were conducted for strain-controlled conditions ($\varepsilon_{at}$ = const.) and stress-controlled conditions ($\sigma_{a}$ = const.) in accordance with the guidelines given in ASTM E606-92. The tests were performed on the Intron 8502 testing machine at the temperature T = 20 °C. Five levels of controlled total strain $\varepsilon_{at}$, and of controlled stress $\sigma_{a}$ were applied in tests. Three as-received samples and three pre-strained samples were tested on each load level. Strain levels ($\varepsilon_{at}$) were adopted after analyzing the static tensile diagrams (see Figure 2), while stress levels $\sigma_{a}$ were determined after low-cycle fatigue tests in the conditions $\varepsilon_{at}$ = const. In the absence of a stabilization period, to establish comparable test conditions, stress amplitudes were assumed based on the middle cycle of each strain-controlled test (n/N = 0.5). The amplitudes of all fatigue tests are listed in Table 2.

Instantaneous stresses during the cyclic loading were obtained as a quotient of the instantaneous values of forces loading the specimen and its initial cross-sectional area. The frequency of the load during the tests was 0.2 Hz. The instantaneous values of the force loading the specimen and its deformation were recorded. Each load cycle was described with 200 points. The criteria for the end of the fatigue test were adopted in accordance with the approach given in the standard ASTM E606-92. Strains were measured by the use of a dynamic test extensometer (type 2630-110) mounted on the gauge part of the specimen, with a base of 10 mm and a measuring range of ±1 mm. The force was measured using a force gauge head (2518-113) with a measuring range of ±125 kN.

## 3. Results

### 3.1. Low-Cycle Fatigue Properties

The results of low-cycle fatigue tests were examined in the context of changes in the stress amplitude $\sigma_{a}$ and plastic strain amplitude $\varepsilon_{ap}$, depending on whether the sample was as-received or pre-deformed. 

**Figure 3:**
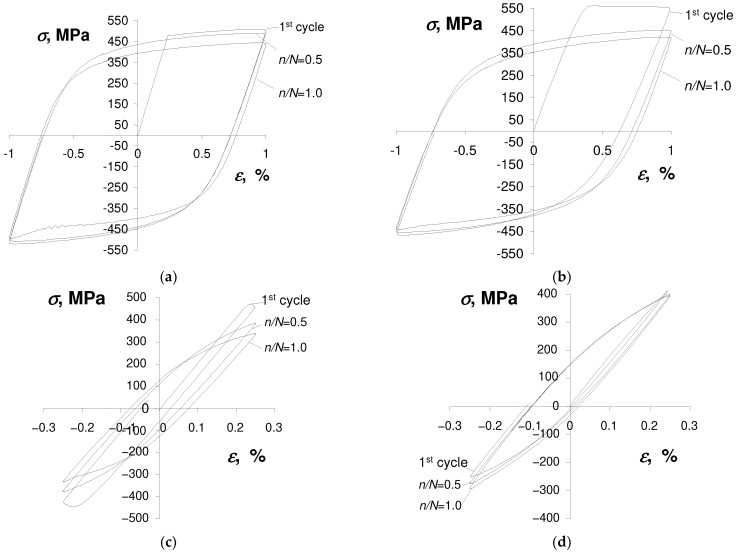
Hysteresis loops for strain-controlled test and strain amplitude levels: (**a**) $\varepsilon_{at}$ = 1.0% (as-received specimen); (**b**) $\varepsilon_{at}$ = 1.0% (pre-strained specimen); (**c**) $\varepsilon_{at}$ = 0.25% (as-received specimen); (**d**) $\varepsilon_{at}$ = 0.25% (pre-strained specimen).

**Figure 4:**
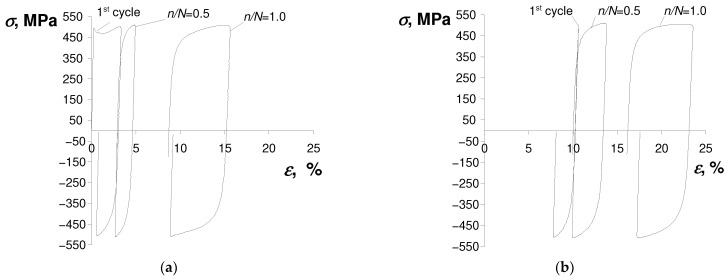
Hysteresis loops for stress-controlled test and stress amplitude level $\sigma_{a}$ = 509 MPa: (**a**) as-received specimen; (**b**) pre-strained specimen.

To illustrate the phenomena observed during the tests, Figure 3 and Figure 4 present exemplary hysteresis loops at two strain amplitude levels of the strain-controlled test (Figure 3) and one stress amplitude level of the stress-controlled experiment (Figure 4) obtained for an as-received specimen and a pre-deformed specimen. The loops were taken from three characteristic durability periods, i.e., the first cycle (n=1), mid-life (n/N=0.5), and the last cycle (n/N=1).

**Figure 5:**
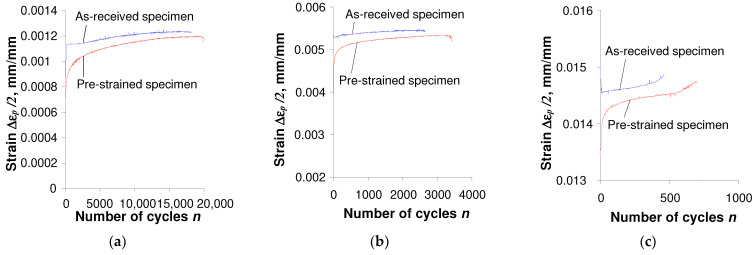
Influence of initial strain on ${∆\varepsilon }_{p}$ observed in strain-controlled tests: (**a**) $\varepsilon_{at}$ = 0.25%; (**b**) $\varepsilon_{at}$ = 0.5%; (**c**) $\varepsilon_{at}$ = 1.0%.

**Figure 6:**
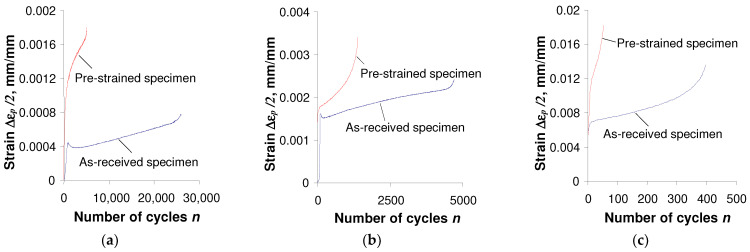
Influence of initial strain on ${∆\varepsilon }_{p}$ observed in stress-controlled tests: (**a**) $\sigma_{a}$ = 384 MPa; (**b**) $\sigma_{a}$ = 430 MPa; (**c**) $\sigma_{a}$ = 509 MPa.

The comparison of test results shown in Figure 3 and Figure 4 confirmed that the hysteresis loop parameters of both as-received and pre-deformed specimens changed during the tests. Also, irrespective of the specimen, there is no clear stabilization period during the cyclic load, which significantly complicates the analysis of test results. As expected, during the tests under the conditions $\sigma_{a}$ = const. (Figure 4), a pronounced creep is visible. It manifests itself in the shift of successive hysteresis loops in the direction of the horizontal axis. Creep takes place both in the tests of as-received and pre-deformed specimens. The magnitude of the loop displacement is influenced by the stress amplitude level $\sigma_{a}$, and increases with increasing stress. Assuming the range of plastic strain $\varepsilon_{ap}$ in the as-received specimen and pre-strain specimen as a measure of changes in low-cycle properties, it can be concluded that in the conditions $\sigma_{a}$ = const., these changes are definitely larger than those observed in the conditions $\varepsilon_{at}$ = const. In order to illustrate changes in loop parameters, Figure 5 and Figure 6 present exemplary comparative $\varepsilon_{ap}$ charts for both as-received and deformed specimens at three levels of strain amplitude $\varepsilon_{at}$, and corresponding stress amplitude levels $\sigma_{a}$ (see Table 2).

Attention is drawn to the asymmetry of stresses and plastic deformations of specimens subjected to initial deformations at the deformation level $\varepsilon_{at}$ = 0.25%. (Figure 2d). The analysis showed that the level of strain significantly influences the asymmetry of stress. The highest values of average stress $\sigma_{m}$ were obtained at the strain level $\varepsilon_{at}$ = 0.25%. No such phenomenon was found when testing as-received samples (the value of $\sigma_{m}$ was often close to the force measurement error). A detailed analysis of stress asymmetry in the conditions of controlled deformation is included in [10].

The analysis of Figure 5 and Figure 6 shows that during cyclic loads, the plastic strain $\varepsilon_{ap}$ of as-received specimens is significantly different from the corresponding plastic strain of pre-deformed specimens. Based on the comparative study of plastic strain $\varepsilon_{ap}$, it can be concluded that under the conditions $\varepsilon_{at}$ = const. (Figure 5), after permanent pre-strain (ε = 10%), plastic strains are smaller than those observed during testing of as-received specimens. The situation is different when testing in conditions $\sigma_{a}$ = const. Significantly higher strain values $\varepsilon_{ap}$ were observed after permanent deformation at the same stress levels. The results of fatigue tests under various load conditions were developed in accordance with the standard [33]. 

The analytical dependency of stress amplitude $\sigma_{a}$ on plastic strain amplitude $\varepsilon_{ap}$, is described by the equation of the form proposed in [33]:

(1) $$\log\sigma_{a}={\log K}^{\prime }+n^{\prime }\log\varepsilon_{ap}$$

The values of $\sigma_{a}$ and $\varepsilon_{ap}$ hysteresis loop parameters were obtained by means of the least squares method, evaluating the coefficients and exponents of a simple regression described by Equation (1). The results of fatigue life were presented in the $2N_{f}-\varepsilon \;$ coordinate system. Fatigue graphs in the bi-logarithmic scale were approximated by the equation of the following form [33]:

(2) $$\frac{\Delta \varepsilon_{t}}{2}=\frac{\Delta \varepsilon_{e}}{2}+\frac{\Delta \varepsilon_{p}}{2}=\frac{\sigma_{f}^{\prime }}{E}{\left(2N_{f}\right)}^{b}+\varepsilon_{f}^{\prime }{\left(2N_{f}\right)}^{c}$$

**Figure 7:**
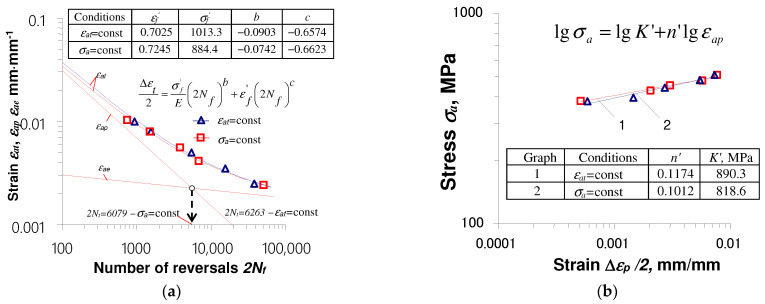
As-received material test results for $\varepsilon_{at}$ = const. and $\sigma_{a}$ = const.: (**a**) fatigue diagrams; (**b**) stress–strain diagrams.

Test results were analyzed in terms of the impact of load conditions ($\sigma_{a}$ = const. or $\varepsilon_{at}$ = const.) and initial strains on the durability and low-cycle properties of S420M steel. It can be concluded that in the conditions $\sigma_{a}$ = const. the fatigue life is slightly lower than the durability obtained in the conditions $\varepsilon_{at}$ = const. To illustrate the results, Figure 7 shows fatigue diagrams described by Equation (2) and a stress–strain diagram described by Equation (1) for the as-received material.

Based on the above results, it was found that the differences in the fatigue life under the conditions $\sigma_{a}$ = const. and $\varepsilon_{at}$ = const. increase with the increase in strain (or stress) amplitude. These results confirm the conclusions presented in [6,7]. 

**Figure 8:**
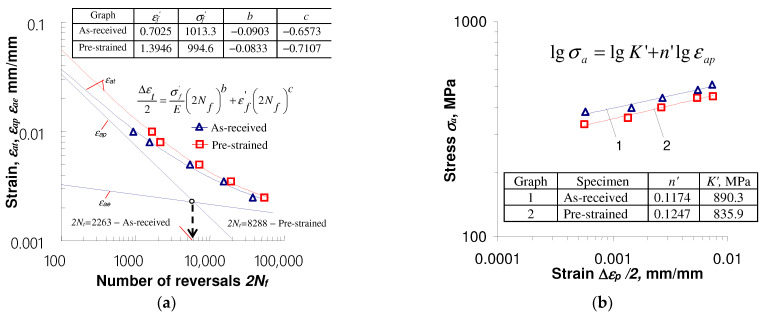
Results of tests performed under strain control $\varepsilon_{at}$ = const.: (**a**) $\varepsilon =f(2N_{f})$; (**b**) $\sigma_{a}=f(\varepsilon_{ap})$.

**Figure 9:**
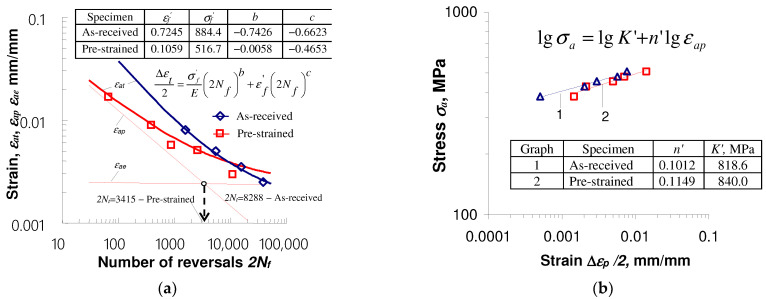
Results of tests performed under stress control $\sigma_{a}$ = const.: (**a**) $\varepsilon =f(2N_{f})$; (**b**) $\sigma_{a}=f(\varepsilon_{ap})$.

Pre-deformation of the specimens affects the low-cycle properties both in stress-controlled and strain-controlled test conditions. Figure 8 and Figure 9 show diagrams illustrating the results of tests carried out in accordance with [33] under conditions $\varepsilon_{at}$ = const. and $\sigma_{a}$ = const.

Based on the test results in the conditions $\varepsilon_{at}$ = const., it can be concluded that at the same strain amplitude levels $\varepsilon_{at}$, the number of cycles to failure of specimens subjected to initial deformation is always larger than that of as-received specimens. It is a consequence of the decrease in stress amplitude $\sigma_{a}$ and plastic strain amplitude $\varepsilon_{ap}$ caused by the initial straining of the specimens (Figure 5). The above conclusion is consistent with the material response observed during the fatigue tests of the TRIP multi-phase steel described in [16]. At lower strain amplitude $\varepsilon_{at}$ levels, the increase in fatigue life of pre-strained specimens is more pronounced. With the increase in strain amplitude, the diversity of the obtained durability decreases. Detailed information on the impact of initial strains on the durability of S420M steel specimens under controlled strain conditions is described in [10] and will not be repeated here.

In the case of tests carried out under conditions of controlled stress $\sigma_{a}$ = const., the durability of pre-deformed specimens is significantly lower than that of as-received specimens (Figure 9). The remarkably higher plastic strains $\varepsilon_{ap}$ of pre-strained specimens at the same stress amplitude levels (Figure 6) result in the reduction in their durability.

**Figure 10:**
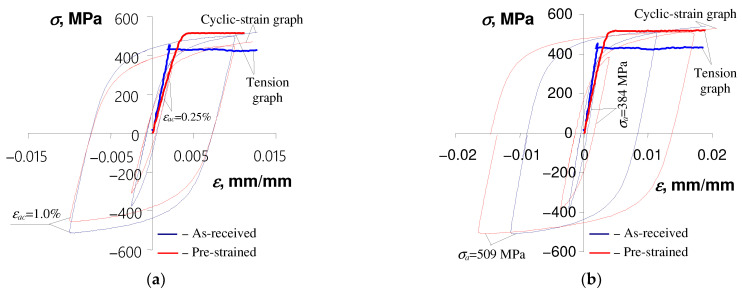
Effect of pre-strain on hysteresis loop characteristics: (**a**) $\varepsilon_{at}$ = const., (**b**) $\sigma_{a}$ = const.

The above results conclude that the basic fatigue characteristics of S420M steel as-received and pre-deformed specimens differ. For example, Figure 10 presents diagrams of cyclic strain approximated by the Ramberg–Osgood equation in the following form:

(3) $$\varepsilon_{at}=\frac{\sigma_{a}}{E}+{\left(\frac{\sigma_{a}}{K^{\prime }}\right)}^{\frac{1}{n^{\prime }}}$$

The hysteresis loops at half-life (n/N=0.5), obtained at two load levels, are shown in Figure 10 for comparison of pre-deformed and as-received specimens’ cyclic properties. The loops obtained in the conditions of $\sigma_{a}$ = const. (Figure 10b) were translated to the origin of the coordinate system.

The location of the cyclic deformation diagrams described by Equation (3) confirms the change in the cyclic properties of the S420M steel specimens as a result of the initial strain. 

Based on the analysis of the mutual location of monotonic tension diagrams and cyclic diagrams for as-received specimens shown in Figure 10, it can be concluded that the cyclic properties of S420M steel, regardless of the type of load ($\varepsilon_{at}$ = const. and $\sigma_{a}$ = const.), depend on the level of deformation. In the range of deformations of approximately $\varepsilon_{at}$ > 0.5%, the steel is slightly strengthened. This is evidenced by the location of cyclic graphs above monotonic tensile graphs in this area. For strain levels $\varepsilon_{at}$ < 0.5%, as-received specimens are subject to softening. Such properties of as-received specimens were also confirmed in the work [10] on the basis of the analysis of the stress amplitude variation curves. In the case of pre-deformed specimens, the cyclic strain amplitude diagram described by Equation (3) is placed below the static tensile diagram, which confirms that this material exhibits softening over the entire range of deformation.

**Figure 11:**
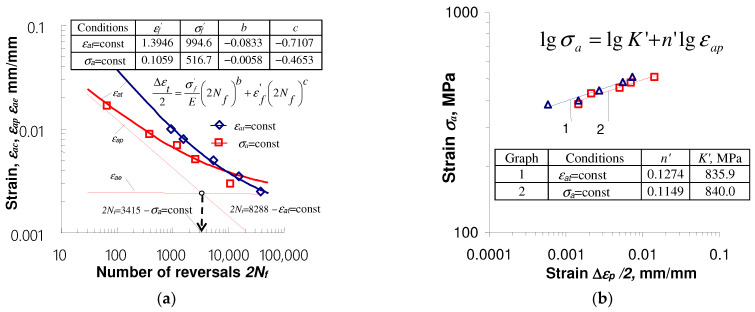
Effect of pre-strain on fatigue properties of S420M steel: (**a**) $\varepsilon =f(2N_{f})$; (**b**) $\sigma_{a}=f(\varepsilon_{ap})$.

Figure 11 shows comparative fatigue diagrams obtained for pre-deformed specimens under two types of loading schemes.

Summarizing the results of tests for pre-deformed and as-received specimens, it can be concluded that the impact of pre-deformation on durability depends on the fatigue load conditions that take place after pre-straining. In the case of fatigue stress-controlled tests ($\sigma_{a}$ = const.), a significant reduction in fatigue life takes place. The fatigue life obtained under these conditions is definitely lower than the durability observed in the strain-controlled ($\varepsilon_{at}$ = const.) fatigue tests. This observation is of great practical importance. It indicates that disregarding the unfavorable effect of pre-straining on durability in conditions $\sigma_{a}$ = const. may result in erroneous predictions of structural element durability.

### 3.2. Microstructural Observations

The microstructure of S420M steel in the as-received state, as well as its modification due to static tensile test, were described in [10]. 

**Figure 12:**
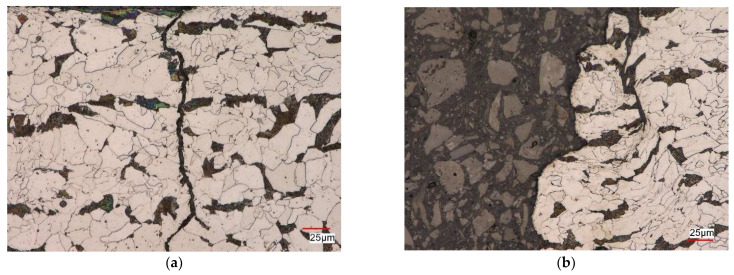
S420M steel microstructure after fatigue test: (**a**) $\varepsilon_{at}$ = const. − $\varepsilon_{at}$ = 1.0%; (**b**) $\sigma_{a}$ = const. − $\sigma_{a}$ = 509 MPa.

To compare the influence of the test control scheme on the microstructure of non-pre-strained S420M steel, Figure 12 shows the microstructure of the steel after the fatigue test of the as-received specimen under the strain control $\varepsilon_{at}$ = const. (Figure 12a) and stress control $\sigma_{a}$ = const. (Figure 12b).

**Figure 13:**
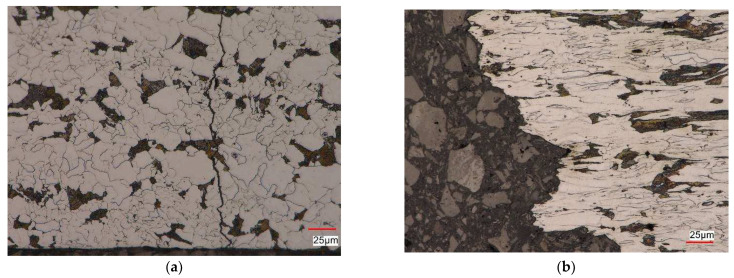
S420M steel microstructure of pre-strained specimen (ε= 10%) after fatigue test: (**a**) $\varepsilon_{at}$ = const. − $\varepsilon_{at}$ = 1.0%; (**b**) $\sigma_{a}$ = const. − $\sigma_{a}$ = 509 MPa.

In the case of as-received specimens subjected to the cyclic strain-controlled load $\varepsilon_{at}$ = const., a ferritic–pearlitic microstructure with characteristics similar to the material in the delivery condition was observed [10] near the fracture site (Figure 12a). The micro-damage in the specimen developed along the ferrite and pearlite grain boundaries and grew in the direction perpendicular to the axis of the specimen. Cracks that propagated parallel to the scrap surface were also detected. Fracture of the specimens subjected to stress-controlled cyclic load ($\sigma_{a}$ = const.) occurred as a result of the nucleation of cracks parallel and perpendicular to the surface of the scrap in the pearlite areas. The propagation of cracks followed the grain boundaries with their local grain deformation (Figure 12b). Figure 13 presents an example of the microstructure obtained after fatigue tests of a pre-strained specimen at the strain amplitude level $\varepsilon_{at}$ = 1.0% (Figure 13a), and the stress amplitude level $\sigma_{a}$ = 509 MPa (Figure 13b). In the case of the strain-controlled test (Figure 13a)—the microstructure in the vicinity of the scrap surface was similar to the material in the delivery condition. The micro-damage nucleated on the side surface of the sample and spread both along the grain boundaries and inside the grains. Initially, cracks were observed parallel to the scrap surface, as in the case of samples made from the as-received material. In the area of decohesion of the tested material, a strongly deformed, banded ferritic–pearlitic microstructure with grains elongated in the direction of the principal stress was observed. The microstructure also revealed the presence of quite numerous nucleating voids in the pearlitic areas as well as in the ferrite (Figure 13b).

**Figure 14:**
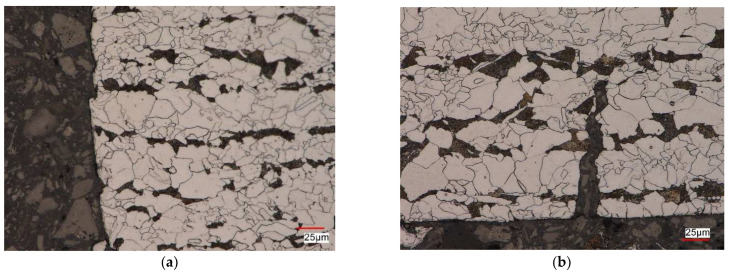
S420M steel microstructure of pre-strained specimen (ε= 10%) after fatigue test: (**a**) $\varepsilon_{at}$ = 0.35%, $\varepsilon_{at}$ = const.; (**b**) $\sigma_{a}$ = 384 MPa − $\sigma_{a}$ = const.

Figure 14 presents the microstructure of a pre-strained S420M steel specimen after fatigue tests for the strain-controlled case at the strain amplitude level $\varepsilon_{at}$ = 0.35% (Figure 14a) and for stress-controlled scheme at the stress amplitude level $\sigma_{a}$ = 384 MPa (Figure 14b).

For both test control schemes, a ferritic–pearlitic microstructure with characteristics similar to the material in the delivery state was observed near the surface of the scrap. In the regions near the surface of the scrap, cracks propagating in the directions parallel to the surface were revealed. The material damage occurred on the side surface of the specimen, where cracks of various depths were revealed, propagating along the grain boundaries perpendicularly to the axis of the specimen (Figure 14b). On the other hand, on the surface of the scrap, a ferritic–pearlitic microstructure was visible, locally deformed, and characterized by a revealed substructure. Studies of the microstructure of S420M steel showed that in most cases, regardless of the assumed deformation and stress parameters, the fracture of the steel was brittle. The nucleation of micro-cracks in these cases was observed on the side surfaces of the specimens, and the propagation took place both along the boundaries and inside the grains. For micro-cracking preceded by plastic deformation (for the case $\sigma_{a}$ = 509 MPa—initial deformation ε = 10%), material damage was associated with the appearance of micro-voids in the microstructure.

## 4. Conclusions

The low-cycle fatigue tests of the S420M steel specimens performed within the present research allow for the formulation of the following conclusions:

1. Loading conditions (test control scheme) affect the fatigue life of both as-received and pre-strained specimens. The fatigue life obtained in the stress control conditions ($\sigma_{a}$ = const.) is lower than the durability observed in the strain control conditions ($\varepsilon_{at}$ = const.). One factor influencing the variation in durability is creep accompanying the cyclic load under stress control conditions. These results confirm findings published in [6,7].
2. Comparative analysis of the hysteresis loop basic parameters of the as-received and pre-deformed S420M steel specimens, obtained at the same strain and stress amplitude levels, reveals that pre-straining causes a significant change in the fatigue properties.
3. Initial deformation preceding variable load under $\varepsilon_{at}$  = const. conditions causes a slight increase in fatigue life. It is caused by the reduction after permanent deformation of two basic parameters of the hysteresis loop, i.e., plastic strain $∆\varepsilon_{p}$, and stress amplitude $\sigma_{a}$.
4. Initial deformations of the specimens (ε = 10%) cause stress asymmetry and plastic deformation during variable loading under the conditions $\varepsilon_{at}$ = const. The value of the average stress $\sigma_{m}$ and plastic strain is influenced by the level of deformation, $\varepsilon_{at}$. The average stress $\sigma_{m}$ reaches its highest values at the lowest level of deformation ($\varepsilon_{at}$ = 0.25%).
5. Pre-straining preceding a cyclic load under controlled stress conditions ($\sigma_{a}$ = const.) results in a reduction in fatigue life. It is caused by a much larger range of plastic deformations in pre-strained specimens in relation to as-received specimens.
6. The fatigue life calculations may be carried out only if the fatigue diagram and the loading program are known. It is also necessary to adopt a damage summation hypothesis, e.g., Palmgren–Miner. This issue was not considered in this paper. The presented research showed that initial deformations of the material might cause a change in its fatigue properties, described using a fatigue diagram. Neglecting this issue during fatigue calculations of pre-deformed structural elements and using only the characteristics determined for the as-received material may lead to significant errors in the durability assessments obtained from calculations.

## Figures and Tables

**Table 2:** Test parameters.

Level	T = 20 °C	
	$\varepsilon_{at}$$,\;\%,\;(\varepsilon_{at}$ = const.)	$\sigma_{a}$$,\;\mathrm{MPa},\;\sigma_{a}$ = const.)
1	0.25	384
2	0.35	415
3	0.5	430
4	0.8	480
5	1.0	509

## Data Availability

Data are contained within the article.
